# Supplementary material for: Long-term efficient organic photovoltaics based on quaternary bulk heterojunctions
Source: Nat Commun. 2017 Jan 16;8:14068. doi: 10.1038/ncomms14068 (PMC5241808; doi:10.1038/ncomms14068)
Supplement: Supplementary Information — Supplementary Figures, Supplementary Tables, Supplementary Notes, Supplementary Methods and Supplementary References [file ncomms14068-s1.pdf]

Supplementary Methods

Charge carrier mobility measurement. The hole and electron mobilities of devices with different annealing durations were measured by the space-charge-limited current (SCLC) method using hole-only devices and electron-only devices, respectively. The device structure of the hole-only device was ITO/PEDOT:PSS/active layer/MoO_3/Au, while the electron-only device was composed of ITO/PFN/active layer/Al. The SCLC model is described by Supplementary Equation 1

$$J_{\mathrm{SCLC}} \cong \frac{9}{8}\varepsilon_0\varepsilon_{\mathrm{r}}\mu_0\frac{V^2}{L^3}\exp\left(0.89\sqrt{V/E_0L}\right) \qquad \text{(Supplementary Equation 1)}$$

where ε_0 is the vacuum permittivity (8.85×10^{12} F/m), ε_{r} is the relative dielectric constant of the polymer blends (assumed as 3), μ_0 is the zero-field mobility, L is the thickness of the active layer, and E_0 is the characteristic field constant. The experimentally obtained J–V characteristics were plotted as $\ln(JL^3/V^2)$ $vs.$ $(V/L)^{0.5}$ to obtain μ_0 through the slope and y-intercept value[1].

Raman analysis. The samples for Raman measurement were prepared as they have been for GIWAXS measurement. The Raman measurement was conducted using an inVia Raman microscope (Renishaw). The excitation source was 532 nm laser, and an acquisition time was minimized as 1 sec to reduce the photo-degradation of the polymer-fullerene blends during measurement[2]. In order to interpret the changes in the Raman spectrum at around 1450 cm^{-1}, we performed the first-principle calculations of PTB7 and $PC_{71}BM$ by using the Plane-Wave Self-Consistent Field (PWscf) and PHonon codes of the Quantum ESPRESSO distribution with the projector-augmented wave (PAW) method and Perdew-Burke-Ernzerhof (PBE) exchange-correlation function[3]. The energy cutoff of 50 Rydberg was used for the molecular structure relaxations and the Raman spectrum calculations. Based on the experimental results, two vibrational normal modes were considered at 1457 cm^{-1} and 1441 cm^{-1} for $PC_{71}BM$ and PTB7, respectively.

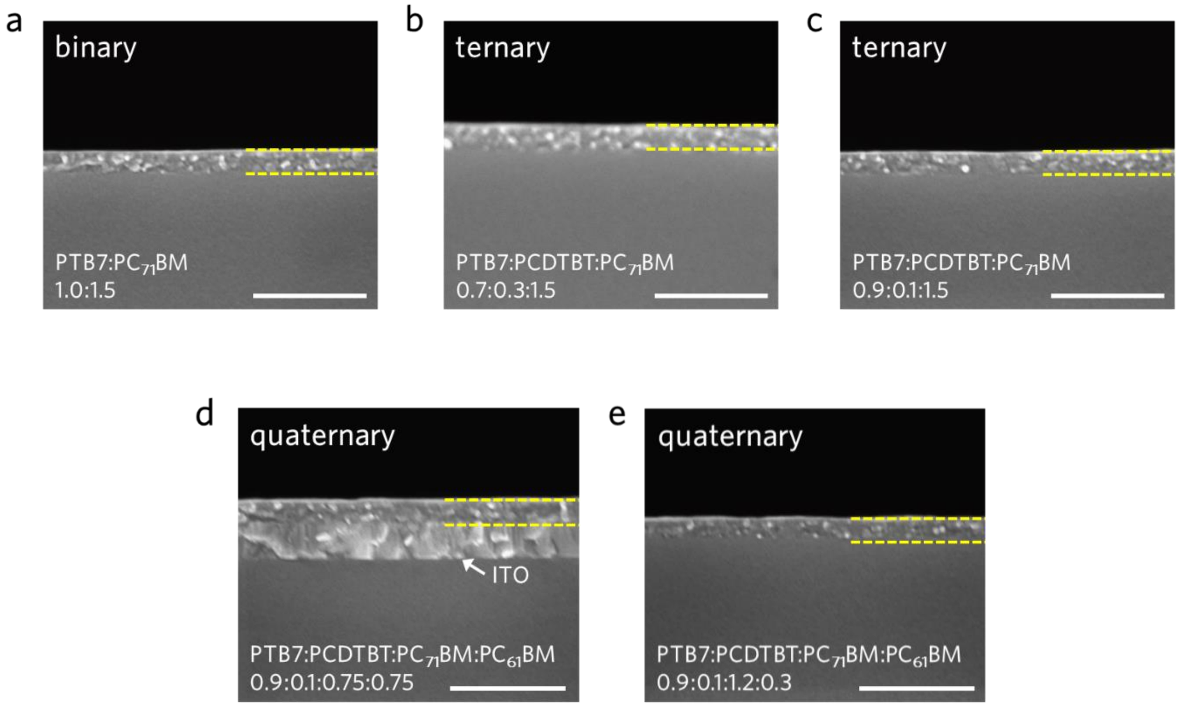

Supplementary Figure 1. Globally fixed active layer thickness. Cross-sectional scanning electron microscopy (SEM) images of active layers (defined with yellow dashed lines) comprised of different compositions: (a) PTB7:$PC_{71}BM$ (1:1.5) binary, (b) PTB7:PCDTBT:$PC_{71}BM$ (0.7:0.3:1.5) ternary, (c) PTB7:PCDTBT:$PC_{71}BM$ (0.9:0.1:1.5) ternary, (d) PTB7:PCDTBT:$PC_{71}BM$:$PC_{61}BM$ (0.9:0.1:0.75:0.75) quaternary, and (e) PTB7:PCDTBT:$PC_{71}BM$:$PC_{61}BM$ (0.9:0.1:1.2:0.3) quaternary. All active layers exhibit a similar film thickness of around 90 nm. The scale bars are 500 nm.

Supplementary Note 1. Transfer process in the blends

As shown in Supplementary Figure 2, the emission intensity from PCDTBT (centered at 705 nm) decreased, while the PTB7 emission (centered at 770 nm) increased when PCDTBT was mixed with PTB7 such that PTB7:PCDTBT = 0.5:0.5. This PL emission change strongly indicates the effective energy transfer from PCDTBT to PTB7[4,5]. On the other hand, when fullerene was mixed in the PTB7:PCDTBT blend (*e.g.*, PTB7:PCDTBT:$PC_{71}BM$ = 0.5:0.5:1.5), the PL emission peaks from both PCDTBT and PTB7 were distinctively quenched. This can be derived by efficient charge transport between polymers and fullerenes (refer to energy band diagram in Figure 1c)[6]. Therefore, the operation of the q-OPV relies on both energy and charge transfer among the donors and acceptors in the BHJ.

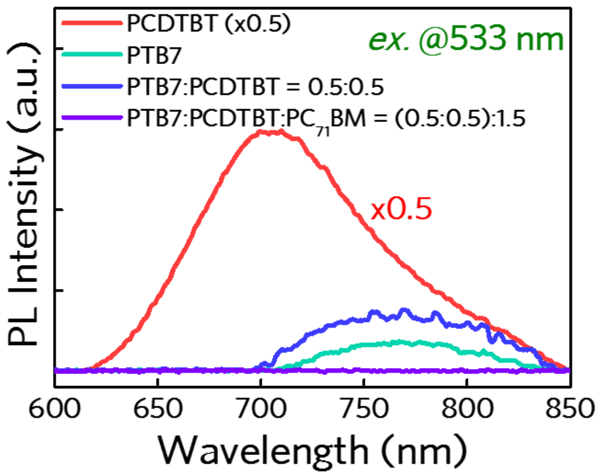

Supplementary Figure 2. Transfer process in the blends. PL intensity spectra of pure PCDTBT, pure PTB7, and binary PTB7:PCDTBT blend (The excitation wavelength of 533 nm corresponds to the main absorption region of PCDTBT). Comparison of these spectra provides evidence of energy transfer from PCDTBT to PTB7. The nearly complete PL quenching in the ternary PTB7:PCDTBT:$PC_{71}BM$ blend indicated efficient charge transfer between the polymers and fullerenes, which is a prerequisite for high-performance OPVs[6].

Supplementary Note 2. Optical response of the OPVs to the solar spectrum

To investigate the optical response of the OPVs to the solar spectrum, we used the T-matrix method followed by calculation of the internal optical E-field distribution in the OPVs consisting of multi-layers[7-9]. Using the optical constants (*i.e.*, n and k values) of the materials[10], absorption (Abs) spectra of the multi-layer stack structure were calculated based on extraction of the transmission (T) and reflection (R) at each of the interfaces between different layers. The T and R values were obtained by calculating series of matrix component composed of the Fresnel complex reflection and transmission at the interface. We then found the average energy dissipation rate $Q_{\mathrm{j}}(x, \lambda)$ in the *j*th layer at incident wavelength λ and at the position x in the thin layers,

$$Q_{\mathrm{j}}(x, \lambda) = \frac{2\pi c \varepsilon_0 k_{\mathrm{j}} n_{\mathrm{j}}}{\lambda} \left| E_{\mathrm{j}}(x)^2 \right| \qquad \text{(Supplementary Equation 2)}$$

where ε_0 is the free space permittivity, subscript j denotes the *j*th layer, c is the speed of light in the free space, and $E_{\mathrm{j}}(x)$ is the optical E-field at the position x. $Q_{\mathrm{j}}(x, \lambda)$ can be translated into the effective absorption of the *j*th layer. Detailed description for the matrix-based modeling of the OPV optical response can be found in literature[7-9]. Supplementary Figure 3a shows the calculated spatial distributions of the squared optical E-field in the thin layers of the q-OPV for different incident wavelengths. Based on the E-field distribution profiles, it was evident that the active layer absorbs most of the incident solar light spanning over the broad range of wavelength (300 - 800 nm), as shown in Supplementary Figure 3b.

Under AM 1.5G condition, it is possible to calculate the exciton generation rate $G(x, \lambda)$, using the energy dissipation rate in the layers as follows:

$$G_{\mathrm{j}}(x, \lambda) = \frac{\lambda}{hc} Q_{\mathrm{j}}(x, \lambda) \qquad \text{(Supplementary Equation 3)}$$

where h is the Planck constant. The total exciton generation rate, $G_{\mathrm{ex}}(x)$, can be calculated such

that $G_{ex}(x) = \int G_j(x, \lambda) d\lambda$. It provides the expected exciton generation rate in the active layer, G_{ex}, and we found that $G_{ex} = \int_0^{90\text{ nm}} G_{ex}(x)\, dx = 1.21 \times 10^{28}\ \text{m}^{-3}\text{s}^{-1}$ for the q-OPV under AM 1.5G condition, which is in a good agreement with the experimentally measured value of $(1.006 \pm 0.224) \times 10^{28}\ \text{m}^{-3}\text{s}^{-1}$ (Supplementary Table 4).

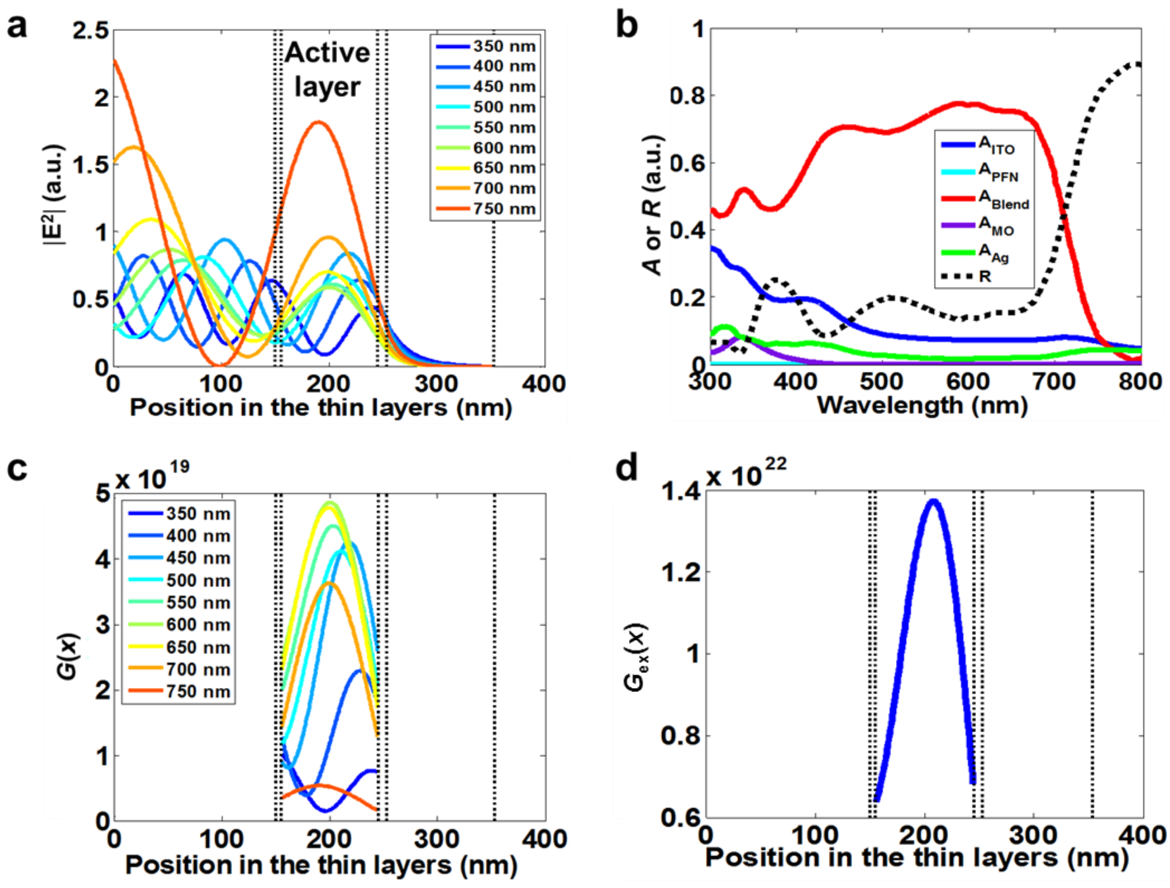

Supplementary Figure 3. Optical calculations for the q-OPV. (a) Spatial distribution of the squared value of the optical E-field ($|E(x)^2|$) in the multi-layered q-OPV for different incident wavelengths. Vertical black dotted lines denote the interface boundary in the thin layered OPV. (b) Optical absorption spectra of the respective layers in the OPV, which delivers the detailed absorption profile of each layer. (c) Spatial distribution of the exciton generation rate ($G(x)$) in the active BHJ layer with

different incident wavelengths assuming an AM 1.5G irradiation condition. The unit of $G(x)$ is number/s·cm^3. (d) Total position-dependent exciton generation rate, $G_{ex}(x)$.

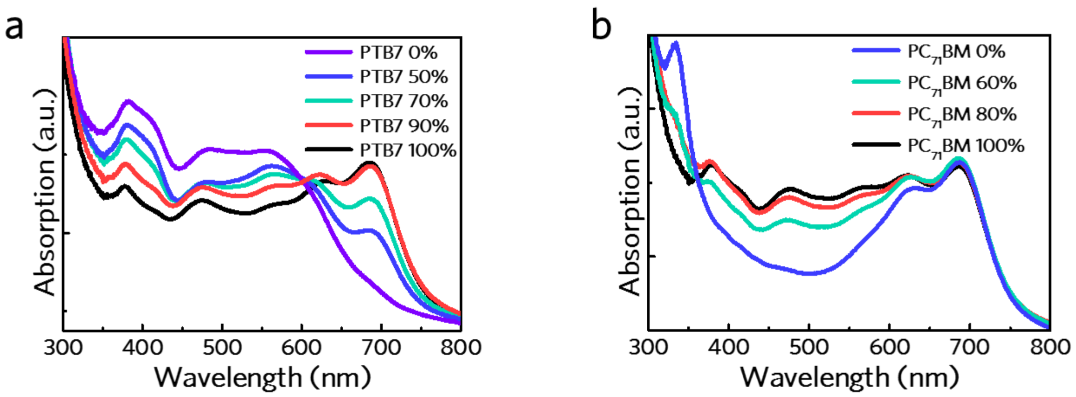

Supplementary Figure 4. Composition-dependent color tunability of the BHJ blends. UV-Vis-NIR absorption spectra of active layers consisting of different D-A BHJ compositions with varying polymer or fullerene concentrations. (a) PTB7:PCDTBT:$PC_{71}BM$ blends with different PTB7 fractions. (b) PTB7:PCDTBT:$PC_{71}BM$:$PC_{61}BM$ blends as a function of $PC_{71}BM$ content with the ratio of PTB7:PCDTBT fixed at 0.9:0.1.

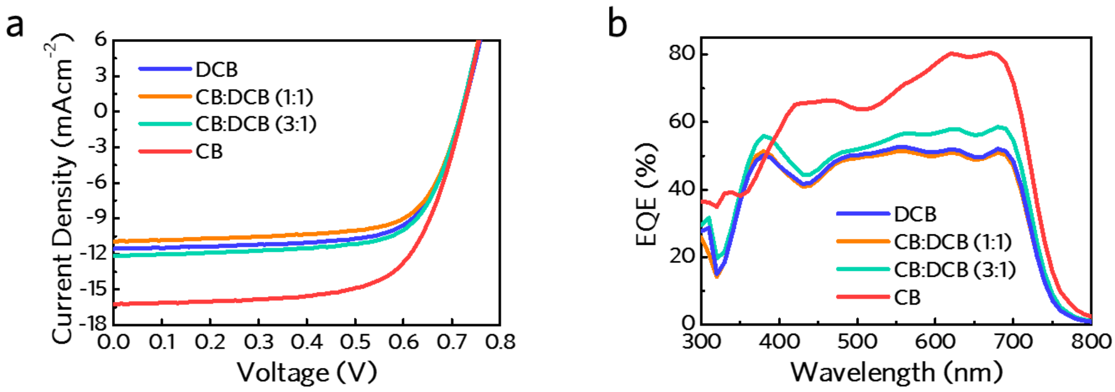

Supplementary Figure 5. Optimizing solvent. (a) *J*–*V* characteristics and (b) EQE spectra of PTB7:$PC_{71}BM$ b-OPV processed with different solvent compositions (CB and DCB denote chlorobenzene and 1,2-dichlorobenzene, respectively).

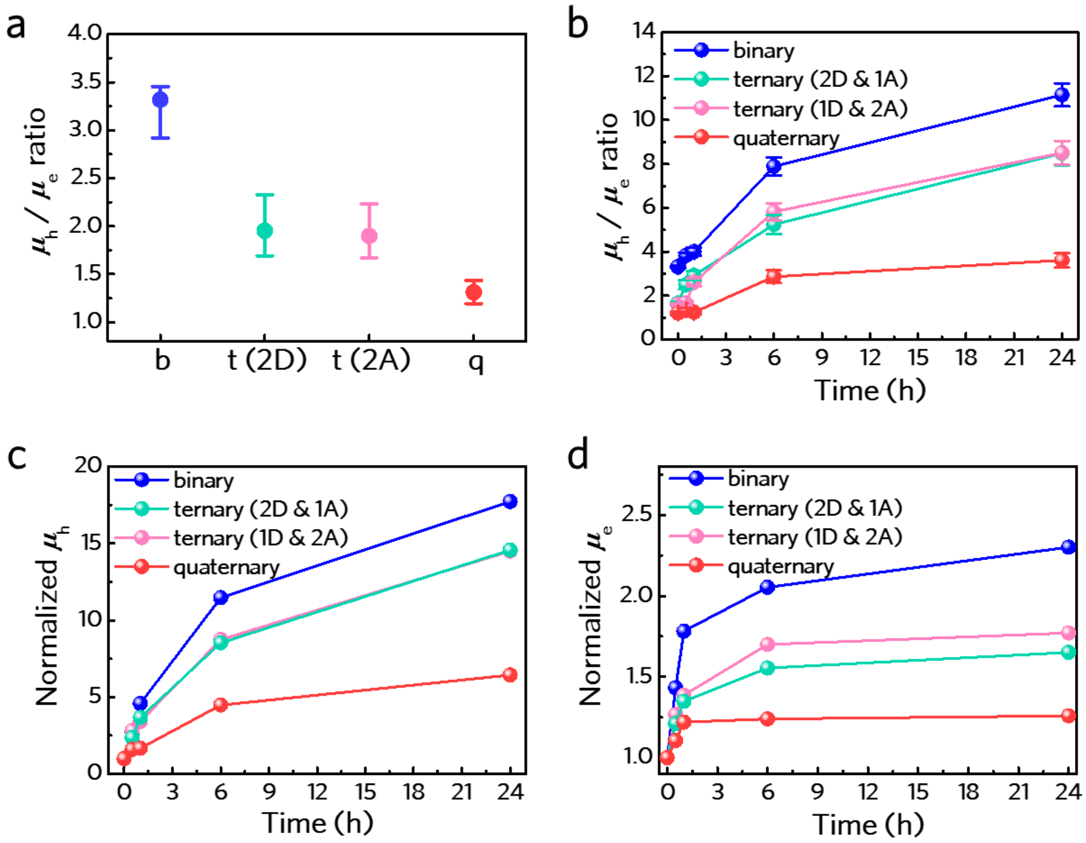

Supplementary Figure 6. Charge carrier mobility. (a) Hole mobility (μ_h) to electron mobility (μ_e) ratios for pristine binary, ternary, and quaternary devices. The t(2D) device was composed of two donors and one acceptor (PTB7:PCDTBT:$PC_{71}BM$ = 0.9:0.1:1.5), while the t(2A) device was composed of one donor and two acceptors (PTB7:$PC_{71}BM$:$PC_{61}BM$ = 1.0:1.2:0.3). (b) The μ_h/μ_e ratio, (c) μ_h, and (d) μ_e at 65ºC for various points up to 24 h. The mean values were obtained from more than 4 devices for each type of sample and the error bars correspond to the standard deviation.

Supplementary Table 1. Comparison of carrier mobility between PTB7:$PC_{71}BM$ binary and PTB7:$PC_{71}BM$:$PC_{61}BM$ 2A ternary devices.

	μ_e [$cm^2V^{-1}s^{-1}$]	μ_h [$cm^2V^{-1}s^{-1}$]	μ_h / μ_e
**PTB7:$PC_{71}BM$**	6.09×10^{-5}	1.99×10^{-4}	3.27
**PTB7:$PC_{71}BM$:$PC_{61}BM$**	9.97×10^{-5}	2.02×10^{-4}	2.02

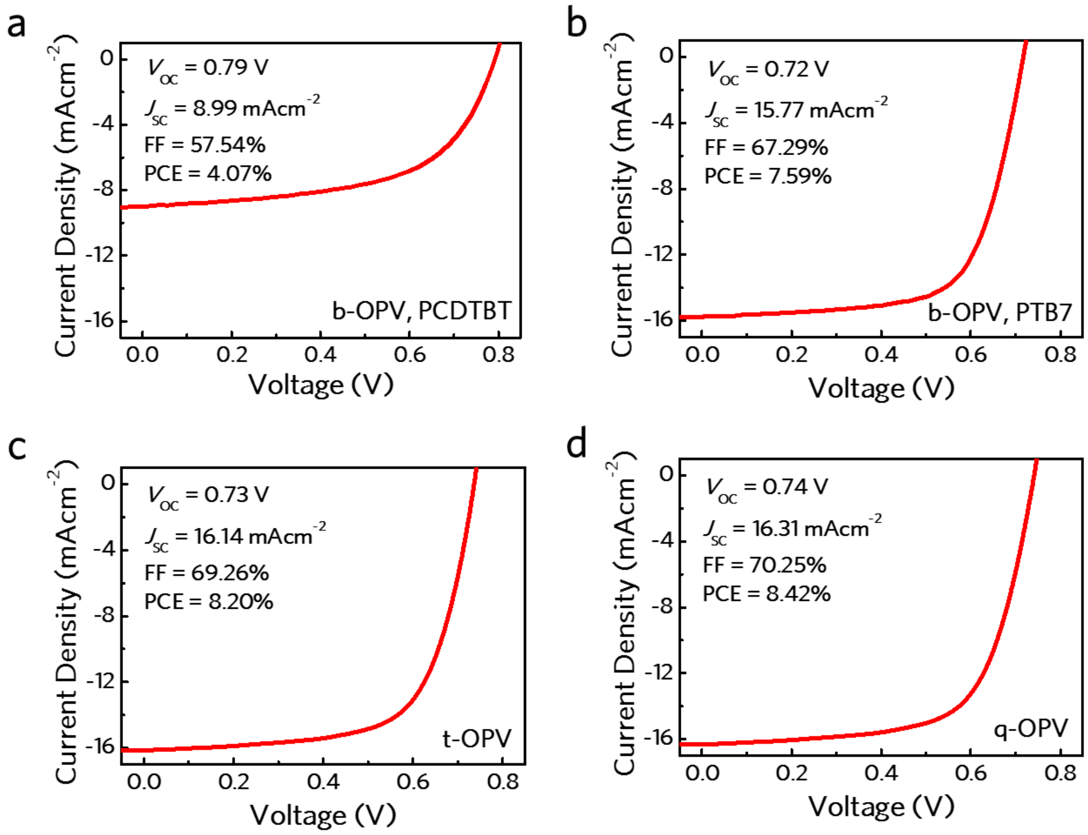

Supplementary Figure 7. PV properties of the pristine OPVs. *J–V* characteristics of (a) PCDTBT-based binary OPV, (b) b-OPV, (c) t-OPV, and (d) q-OPV.

Supplementary Table 2. Summary of active layer composition and corresponding photovoltaic parameters of representative OPVs. The mean values and error ranges corresponding to the standard deviation were obtained from more than 16 cells.

	PTB7	PCDTBT	$PC_{71}BM$	$PC_{61}BM$	V_{OC} (V)	J_{SC} ($mAcm^{-2}$)	FF (%)	PCE (%)
**PCDTBT b-OPV**	–	0.4	1.6	–	0.79 ±0.02	8.99 ±0.16	57.54 ±0.01	4.07 ±0.13
**PTB7 b-OPV**	1.0	–	1.5	–	0.72 ±0.01	15.77 ±0.19	67.29 ±0.01	7.59 ±0.19
**t-OPV**	0.9	0.1	1.5	–	0.73 ±0.01	16.14 ±0.23	69.26 ±0.01	8.20 ±0.08
**q-OPV**	0.9	0.1	1.2	0.3	0.74 ±0.01	16.31 ±0.17	70.25 ±0.02	8.42 ±0.12

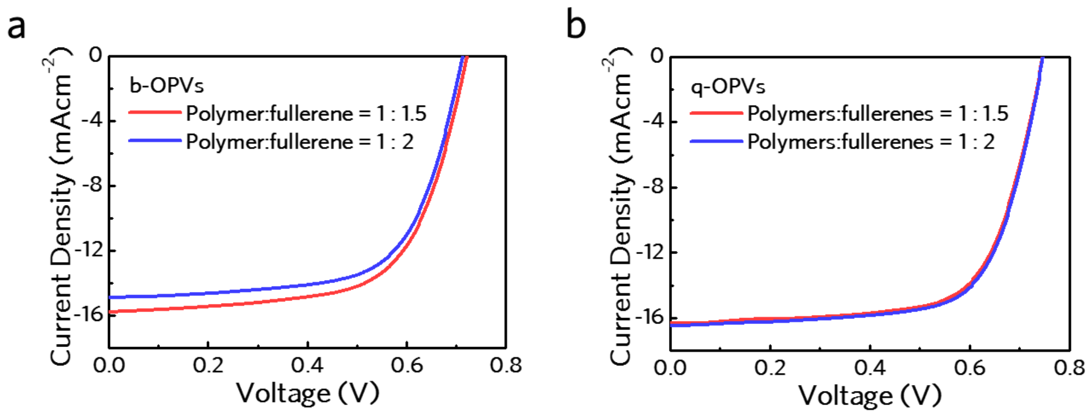

Supplementary Figure 8. D-A ratio-dependent PV properties. *J–V* characteristics of (a) b-OPV and (b) q-OPV as a function of overall polymer(s)-to-fullerene(s) ratio.

Supplementary Table 3. Comparison of the photovoltaic parameters between the b- and q-OPVs with different overall D-A ratios of 1:1.5 or 1:2. The mean values were obtained from at least 4 samples for each type of OPV and the error ranges correspond to respective standard deviation values.

**PTB7:PCDTBT:$PC_{71}BM$:$PC_{61}BM$**	V_{OC} **(V)**	J_{SC} **($mAcm^{-2}$)**	**FF (%)**	**PCE (%)**
**1:0:1.5:0**	0.72 ±0.01	15.77 ±0.19	67.29 ±0.94	7.59 ±0.19
**1:0:2.0:0**	0.71 ±0.01	14.99 ±0.12	67.77 ±0.91	7.18 ±0.23
**0.9:0.1:1.2:0.3**	0.74 ±0.01	16.31 ±0.17	70.25 ±0.56	8.42 ±0.12
**0.9:0.1:1.6:0.4**	0.74 ±0.01	16.46 ±0.13	70.95 ±0.63	8.54 ±0.15

Supplementary Table 4. Summary of parameters associated with capabilities of photon absorption and exciton dissociation in active layers (the parameters were obtained from J_{ph}–V_{eff} characteristics in Figure 2f in the main article).

	PCDTBT b-OPV	PTB7 b-OPV	t-OPV	q-OPV‡
J_{ph} (A m^{-2}, short condition)*	85.7	157.0	161.4	163.1 (± 4.8)
J_{sat} (A m^{-2}, short condition)†	93.1	163.8	169.0	170.0 (± 7.4)
G_{max} (m^{-3} s^{-1})†	6.886×10^{27}	9.692×10^{27}	1.001×10^{28}	1.006×10^{28} (± 0.04×10^{28})
P(E,T) (%)†	92.05	95.85	95.50	97.36 (± 1.41)

*J_{ph} is obtained from $J_L - J_D$.

†J_{sat}, G_{max}, and *P(E,T)* denote the saturation current density, maximum exciton generation rate, and charge dissociation probability, respectively.

‡The average and standard deviation values for the q-OPV were obtained from 4 cells.

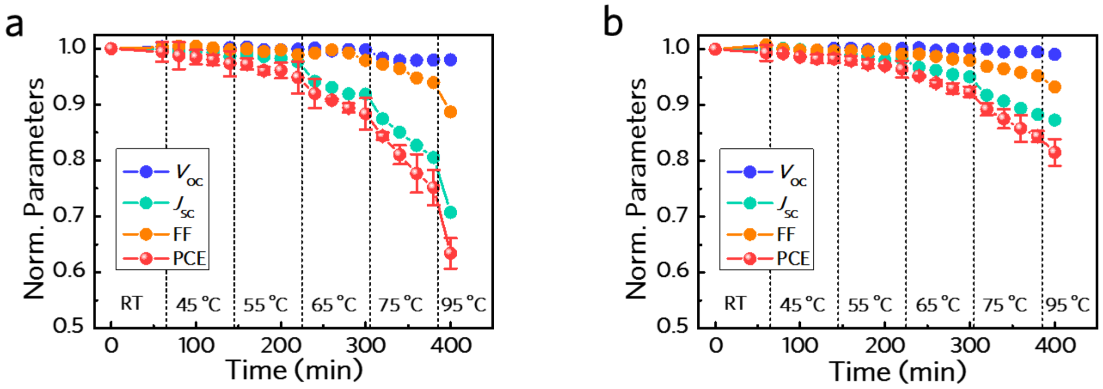

Supplementary Figure 9. PV parameters with varying annealing temperatures. The normalized parameter loss for (a) b-OPV and (b) q-OPV as a function of time and temperature. Device degradation was mainly derived from a loss in J_{SC} as a function of time and was accelerated upon thermal treatment at around 65°C. We presented the average and standard deviation values obtained from at least 4 cells.

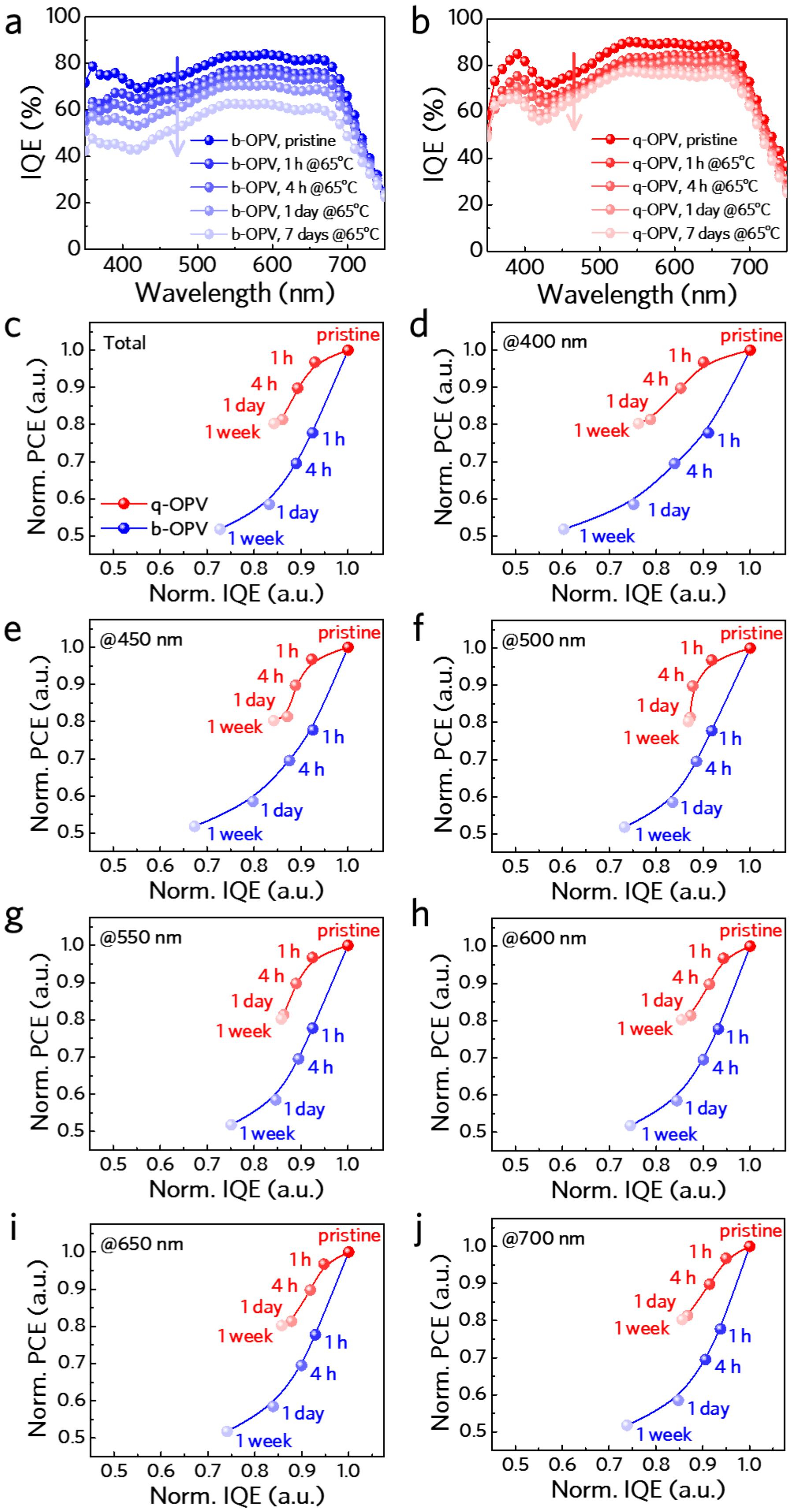
a
IQE (%)
Wavelength (nm)
b-OPV, pristine
b-OPV, 1 h @65°C
b-OPV, 4 h @65°C
b-OPV, 1 day @65°C
b-OPV, 7 days @65°C
b
q-OPV, pristine
q-OPV, 1 h @65°C
q-OPV, 4 h @65°C
q-OPV, 1 day @65°C
q-OPV, 7 days @65°C
c
Total
q-OPV
b-OPV
d
@400 nm
e
@450 nm
f
@500 nm
g
@550 nm
h
@600 nm
i
@650 nm
j
@700 nm
pristine
1 h
4 h
1 day
1 week
Norm. PCE (a.u.)
Norm. IQE (a.u.)

Supplementary Figure 10. IQE transition upon thermal annealing. IQE spectra of (a) b- and (b) q-OPVs with varying thermal annealing durations at 65°C. The IQE spectra were obtained based on IQE = EQE / (1 – R – parasitic absorption), where the experimentally obtained spectral EQE and R values were used. The spectral parasitic absorption data are provided in Supplementary Figure 3b. (c-j) Normalized IQE *vs.* PCE characteristics of b- and q-OPVs at 65°C as a function of incident wavelength and time. These results indicate the PCE to be strongly correlated with the IQE, and the q-OPV exhibited high IQE regardless of the operation time.

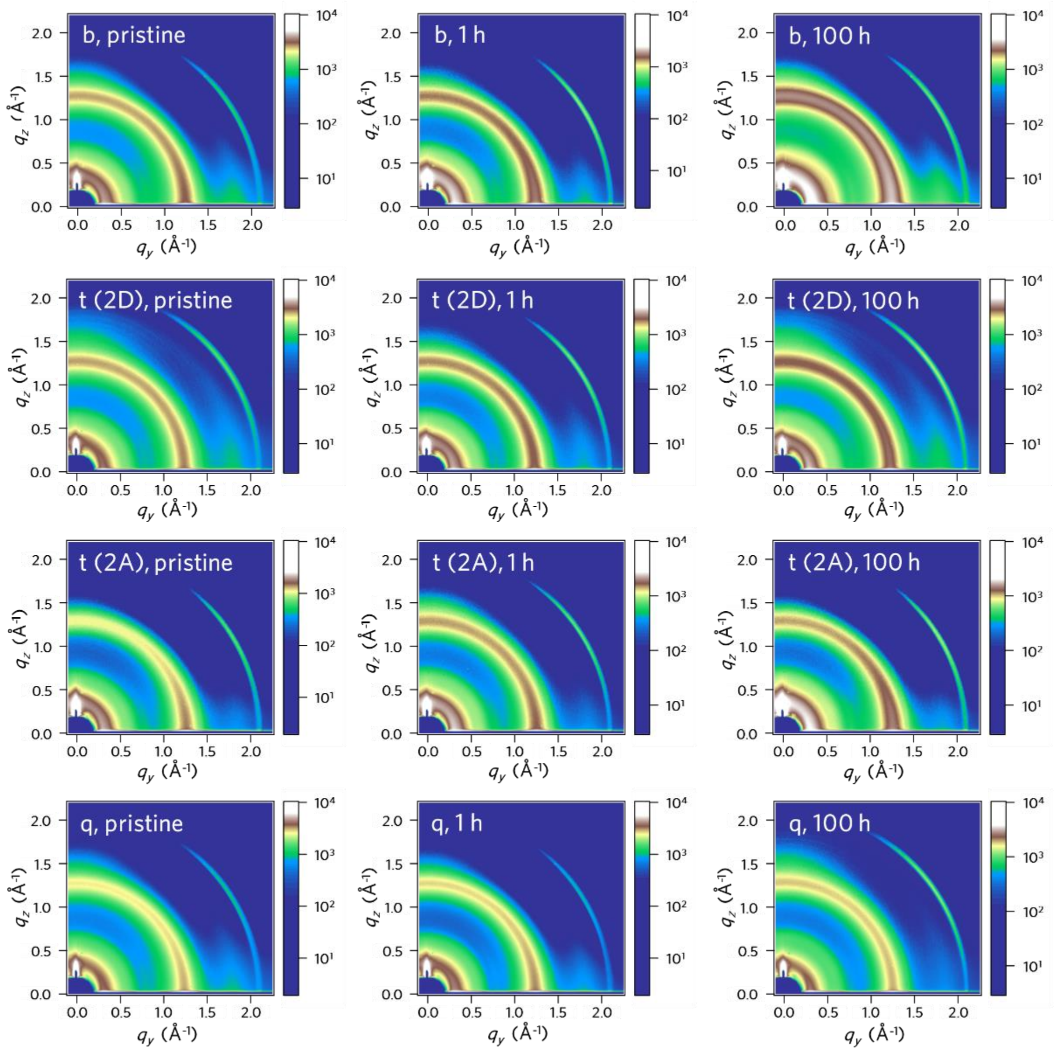

Supplementary Figure 11. GIWAXS analysis. 2D GIWAXS patterns of binary, ternary (2D & 1A or 1D & 2A), and quaternary blends as a function of annealing time at 65°C.

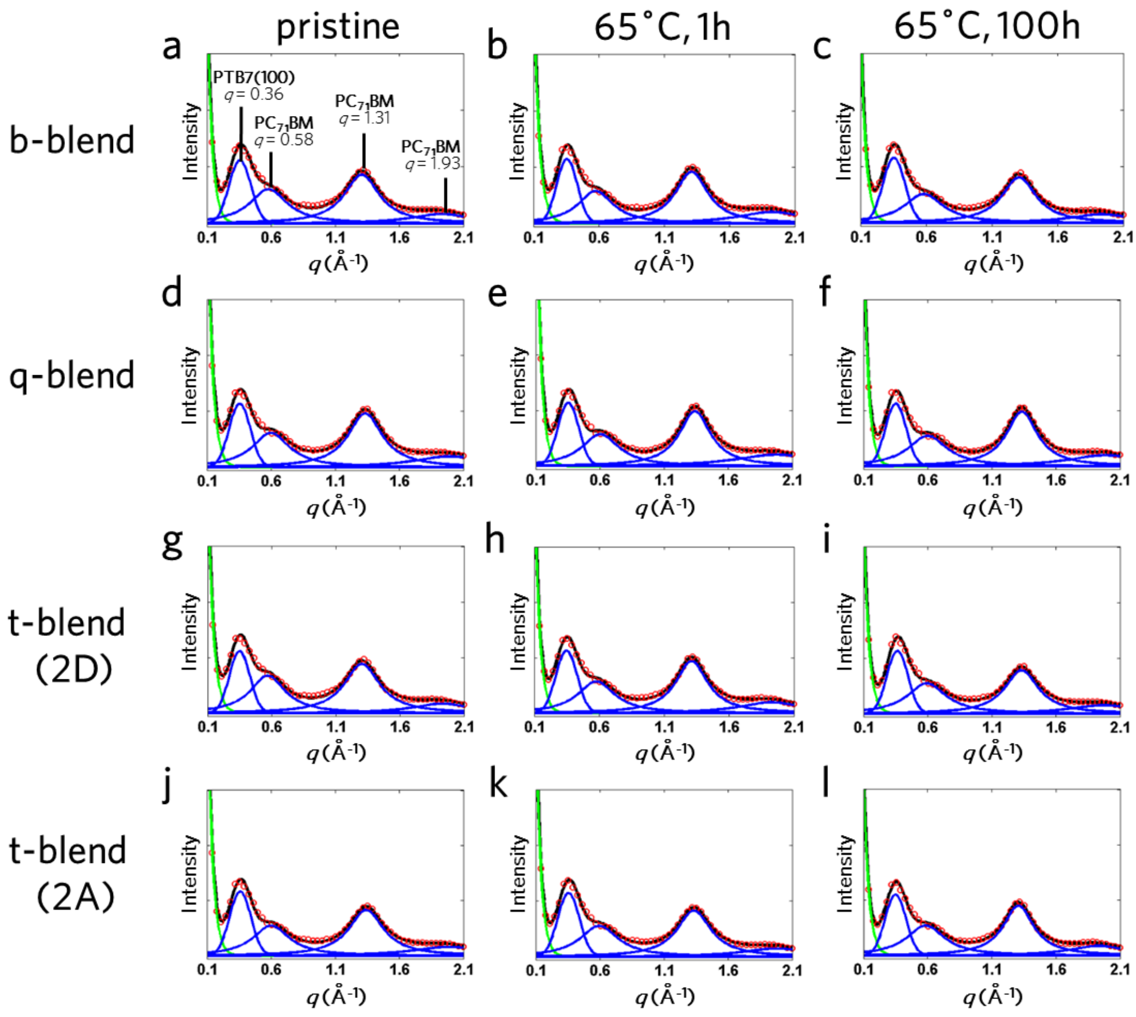

Supplementary Figure 12. Out-of-plane 2D GIWAXS line profiles. Fitting of each of the GIWAXS profiles of the binary, ternary, and quaternary BHJ blends with varying thermal treatment times at 65°C for (a, d, g, and j) 0 h (pristine), (b, e, h, and k) 1 h, and (c, f, i and l) 100 h. The red dotted lines indicate experimental data, black solid lines are fits, blue lines are Lorentz peaks, and green lines are exponential backgrounds.

Supplementary Table 5. Parameters for the 2D GIWAXS profile fits as a function of thermal treatment duration at 65°C.

		PTB7			Fullerene derivatives		
		**Peak ($Å^{-1}$)**	**FWHM ($Å^{-1}$)**	**Crystallite size (Å)**	**Peak ($Å^{-1}$)**	**FWHM ($Å^{-1}$)**	**Crystallite size (Å)**
**b-blend**	**Pristine**	0.36	0.159	37.29	1.31	0.316	19.16
	**1 h**	0.36	0.153	38.78	1.31	0.301	20.15
	**100 h**	0.35	0.142	41.67	1.30	0.284	21.32
**q-blend**	**Pristine**	0.35	0.169	35.07	1.34	0.323	18.76
	**1 h**	0.35	0.163	36.21	1.34	0.32	18.95
	**100 h**	0.35	0.161	36.86	1.33	0.304	19.94
**t-blend (2D & 1A)**	**Pristine**	0.35	0.166	35.64	1.30	0.318	19.04
	**1 h**	0.35	0.160	36.88	1.31	0.302	20.06
	**100 h**	0.35	0.159	37.18	1.31	0.287	21.12
**t-blend (1D & 2A)**	**Pristine**	0.35	0.160	37.06	1.34	0.321	18.86
	**1 h**	0.35	0.154	38.42	1.33	0.314	19.33
	**100 h**	0.36	0.146	40.53	1.33	0.302	20.06

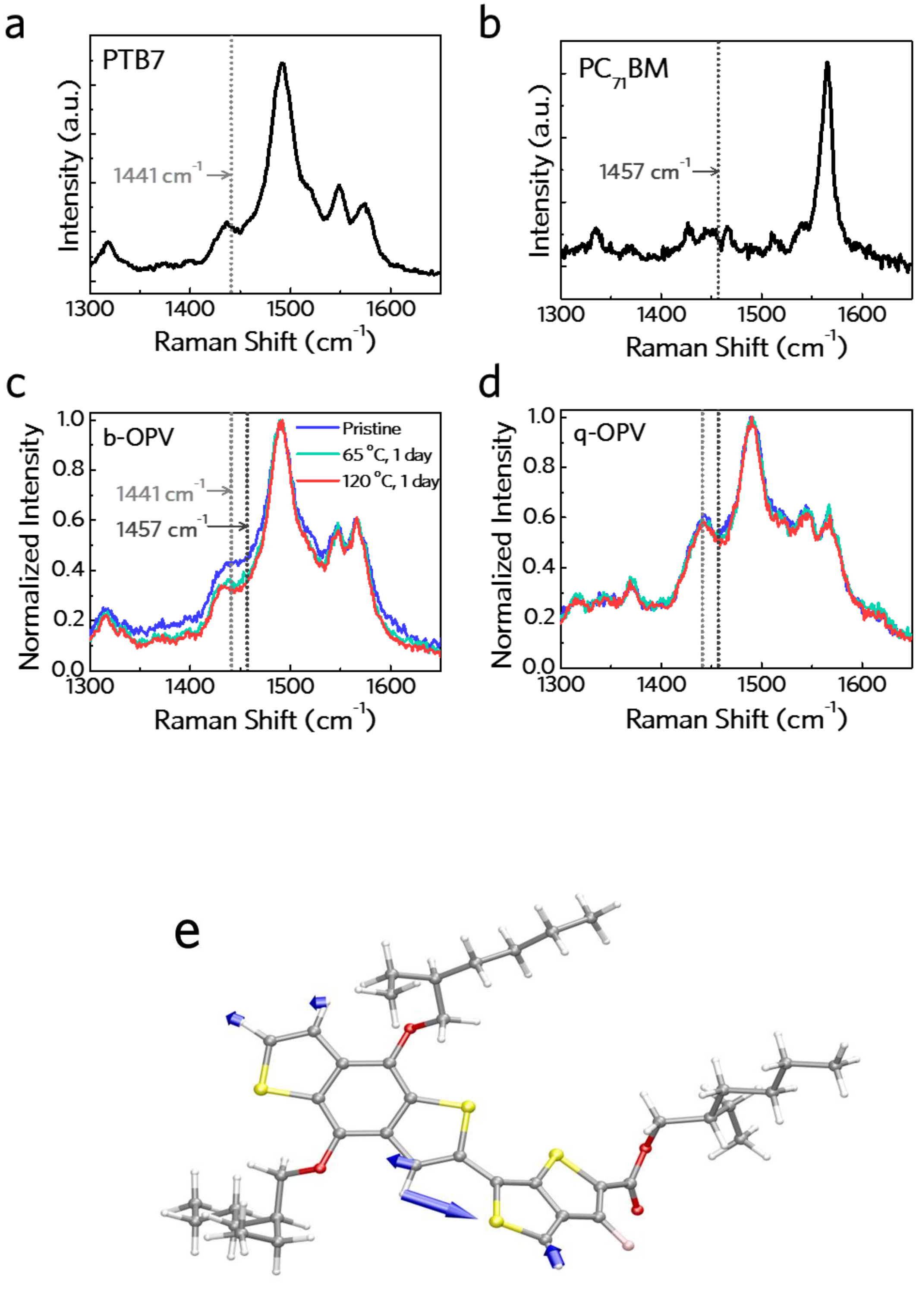

a
PTB7
Intensity (a.u.)
1441 cm^{-1}
1300
1400
1500
1600
Raman Shift (cm^{-1})
b
$PC_{71}BM$
Intensity (a.u.)
1457 cm^{-1}
1300
1400
1500
1600
Raman Shift (cm^{-1})
c
b-OPV
Pristine
65 °C, 1 day
120 °C, 1 day
1441 cm^{-1}
1457 cm^{-1}
Normalized Intensity
0.0
0.2
0.4
0.6
0.8
1.0
1300
1400
1500
1600
Raman Shift (cm^{-1})
d
q-OPV
Normalized Intensity
0.0
0.2
0.4
0.6
0.8
1.0
1300
1400
1500
1600
Raman Shift (cm^{-1})
e

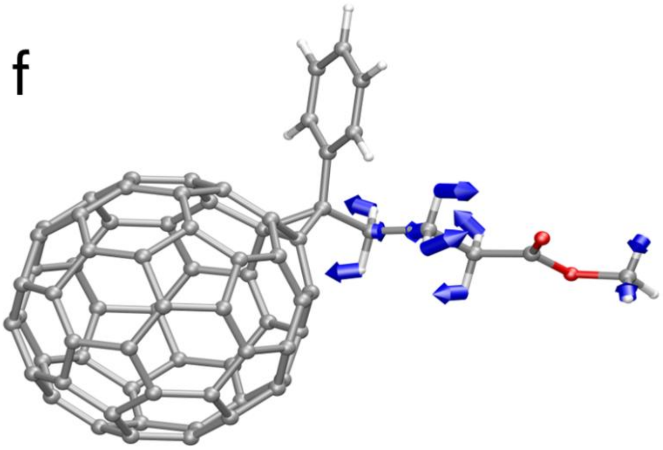

Supplementary Figure 13. Raman analysis. Raman spectra of (a) pure PTB7 and (b) pure $PC_{71}BM$. Transitions in Raman spectra induced by applying thermal annealing for (c) b- and (d) q-blends. Vibrational normal modes of (a) PTB7 at 1441 cm^{-1} and (b) $PC_{71}BM$ at 1457 cm^{-1} obtained from the first-principle calculations. The blue arrows in (e) PTB7 and (f) $PC_{71}BM$ indicate major atomic vibrations for the two modes, where the size and direction of the arrows correspond to the magnitude and direction of atomic vibrations. The dotted vertical lines in (c) and (d) correspond to 1441 cm^{-1} and 1457 cm^{-1} for ease of comparison. The spheres with distinct colors in (e) and (f) represent constituting atoms: carbon (black), hydrogen (white), oxygen (red), sulfur (yellow), and fluorine (purple).

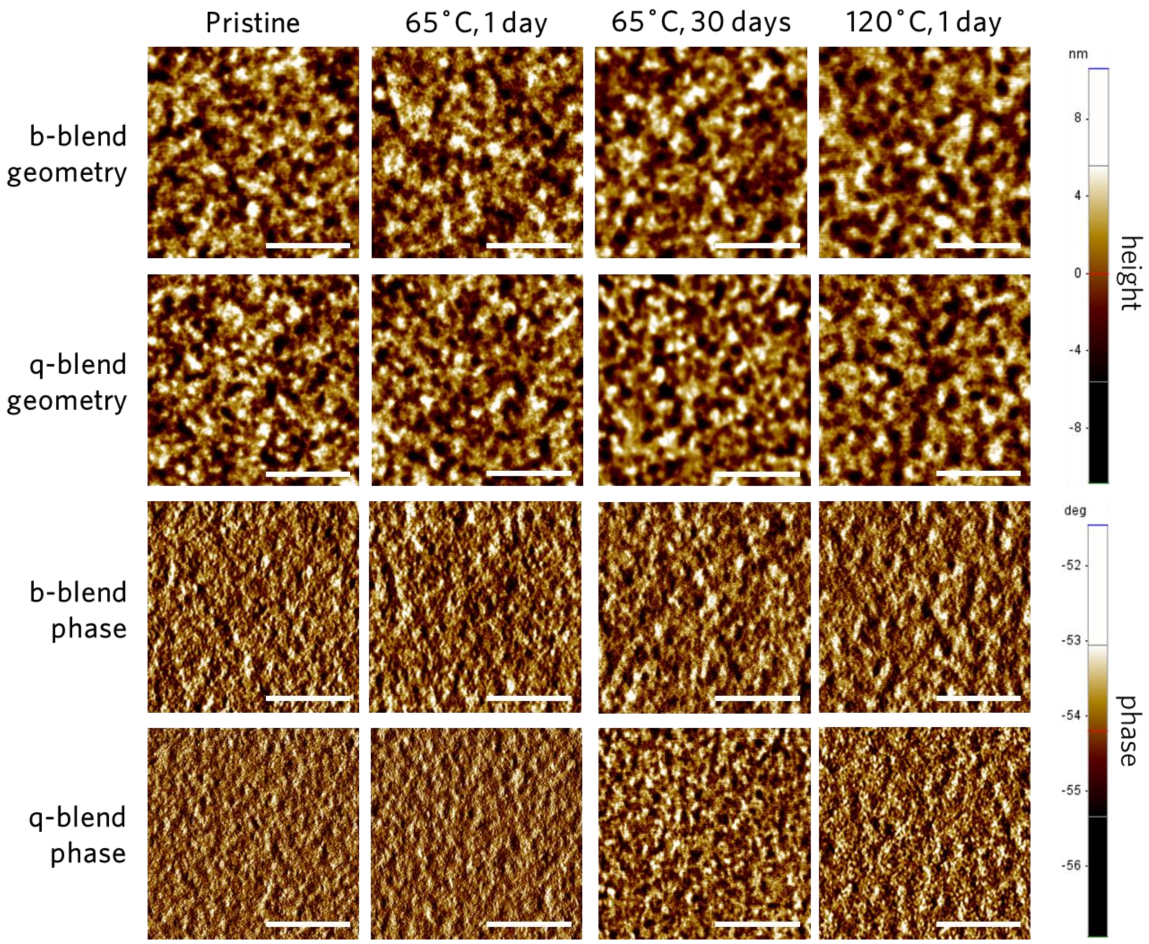

Supplementary Figure 14. AFM analysis. Tapping-mode 2D AFM geometry and phase images of the binary and quaternary blends as a function of annealing temperature and time (scan area was 5 × 5 μm^2 and scales bars denote 2 µm).

Supplementary Table 6. Comparison of L_{cor} and H_{inter} of the b- and q-BHJs operated for 1 day and 30 days at 65°C. The mean values were obtained by AFM analysis of 5 different samples each and the error ranges correspond to the standard deviation.

	Operation duration	$L_{cor,x}$ (nm)	$L_{cor,y}$ (nm)	H_{inter} (nm)
**b-BHJ**	**1 day**	109.9 ± 4.95	250.2 ± 10.26	16.01 ± 0.69
	**30 days**	144.8 ± 6.67	304.1 ± 14.91	20.82 ± 1.00
**q-BHJ**	**1 day**	106.9 ± 3.53	200.3 ± 7.21	15.01 ± 0.53
	**30 days**	137.4 ± 5.08	151.1 ± 4.68	16.18 ± 0.52

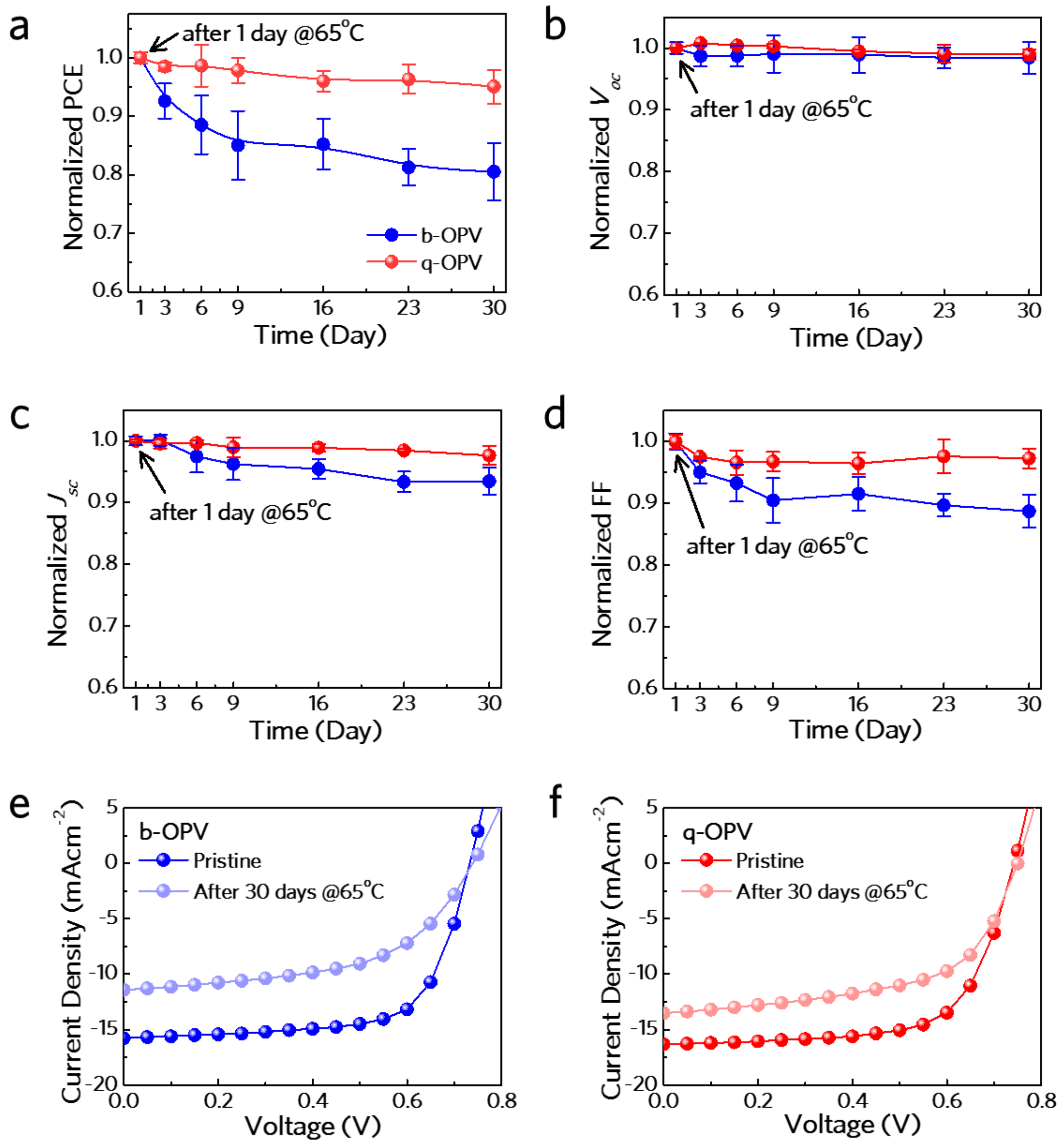

Supplementary Figure 15. Time-dependent OPV performance decay history. (a)-(d) Photovoltaic parameters decay as a function of storage time at 65°C (the devices annealed for one day were assigned as a reference). The average and standard deviation values were obtained from at least 12 cells. *J*–*V* curves of (e) b-OPV and (f) q-OPV before and after thermal treatment at 65°C for 30 days.

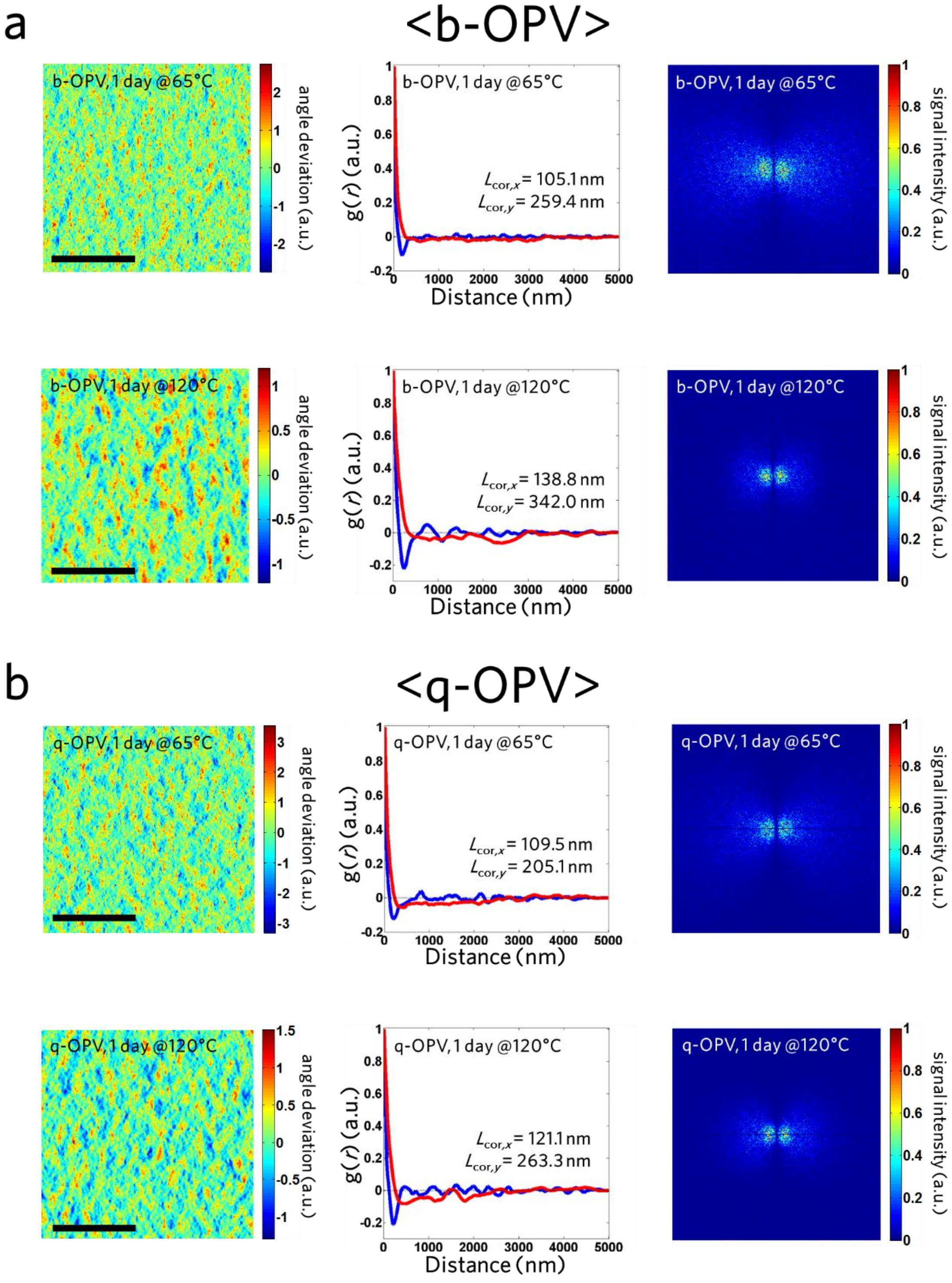

Supplementary Figure 16. Annealing temperature-dependent correlation length scale. (a) A set of 2D AFM phase images with normalized orientational angle deviations, g(r), and 2D FFT profiles

for the (a) b- and (b) q-OPVs with varying annealing temperatures (*i.e.*, 65 vs 120°C) for one day. The AFM scan area was 5 × 5 μm^2 (scale bars denote 2 μm).

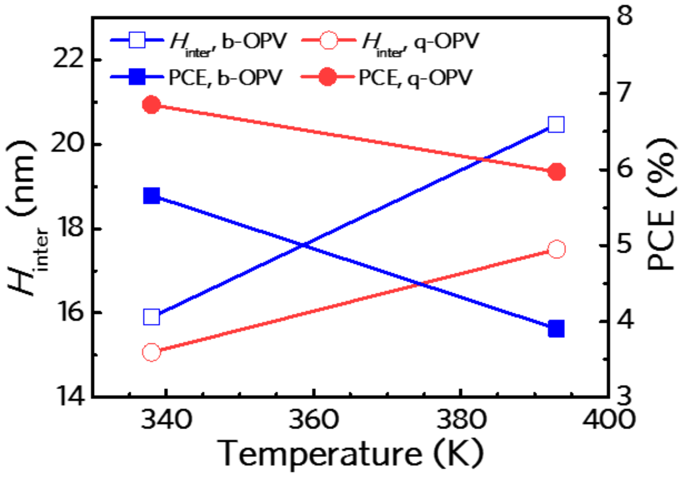

Supplementary Figure 17. Domain growth analysis with varying operating temperatures. Comparison of H_{inter} and PCE of b- and q-OPVs as a function of annealing temperature for one day.

Supplementary Note 3. Theoretical analysis of photovoltaic performances of the OPVs as a function of morphological parameter

For the quantitative study on the effects of the BHJ morphology on photovoltaic performances, we numerically analyzed the charge carrier transport based on volume-averaged drift-diffusion equation and Poisson equation[7-9,11,12]. For simplicity, we assumed that the two donor components have similar chemical affinity. In addition, we also simplified the quaternary blend as a mixture of the donor-rich (D-phase) and acceptor-rich (A-phase) phases in the two-phase spatially smoothed model, in which the donor- and acceptor-rich phases form continuously inter-connected structure in the thin film[11,12]. It was also convenient to introduce the volume-averaged physical entities such as charge carrier flux and concentration, electric potential, and mobilities for respective phases in the two-phase model. Detailed governing equation accompanied by relevant boundary conditions can be expressed as follows:

(1) Electric potential balance

$$\nabla \cdot \left[\varepsilon_0\left(\phi_A \varepsilon_A + \phi_D \varepsilon_D\right) \cdot \nabla\psi\right] = q(\phi_A n_{e,A} - \phi_D n_{h,D}) \qquad \text{(Supplementary Equation 4)}$$

(2) Charge-carrier transport

$$\nabla \cdot \mathbf{J}_e = \nabla \cdot \mathbf{J}_h = pG_{e/h} - \frac{H_{PFM}(1-p)k_r n_{e,A} n_{h,D}}{H_{inter}}, \qquad \text{(Supplementary Equation 5)}$$

$$\text{where } \mathbf{J}_e = -D_e \nabla n_{e,A} + \mu_e n_{e,A} \nabla\Psi,$$

$$\mathbf{J}_h = -D_h \nabla n_{h,D} - \mu_h n_{h,D} \nabla\Psi,$$

$$D_e = \frac{k_B T}{q}\mu_e,\ D_h = \frac{k_B T}{q}\mu_h, \quad \text{and} \quad k_r = \frac{q(\mu_h + \mu_e)}{\varepsilon_0(\phi_A \varepsilon_A + \phi_D \varepsilon_D)}$$

where ϕ is the volumetric fraction with subscripts A and D denoting the acceptor and donor phases, respectively, ε is the dielectric constant, q is the electric charge, $n_{e,A}$ and $n_{h,D}$ are the

concentrations of electrons in the acceptor and holes in the donor phases, respectively, and ψ is the electric potential in the BHJ thin film such that, $\psi = \mathrm{V_A} - \mathrm{V_{built}}$, where $\mathrm{V_A}$ and $\mathrm{V_{built}}$ are the applied bias voltage and the built-in voltage, respectively. For charge-career continuity equation in the second part, the charge-carrier flux, $\mathbf{J}$, is a function of diffusivity D, potential gradient, and mobility, where D can be expressed by the generalized Einstein equation depending on the thermal energy ($k_\mathrm{B}T$) and mobility (μ). The flux gradient is balanced with the charge-carrier generation and recombination, which are governed by probabilistic generation rate, $pG_{\mathrm{e/h}}$, where p denotes the probability of dissociation of electron-hole pairs at the donor-acceptor interfaces, and the recombination rate with the kinetic constant of k_r. The volumetric generation rate of electron-hole pairs at the donor-acceptor interface, $G_{\mathrm{e/h}}$, can be expressed as $G_{\mathrm{e/h}} = -\frac{\mathbf{J}_{\mathrm{ex}} \cdot \hat{\mathbf{n}}_{\mathrm{inter}}}{H_{\mathrm{PFM}}}$, where $\mathbf{J}_{\mathrm{ex}}$ is the flux of the generated exciton in the donor phase, $\hat{\mathbf{n}}_{\mathrm{inter}}$ is the unit vector normal to the donor-acceptor interface, and H_{PFM} corresponds to the interface-depth in the phase field model[13-15], which is nearly constant without respect to the morphology[12,13,16]. $\mathbf{J}_{\mathrm{ex}}$ can be calculated using transport equation of the exciton such that

$$\nabla \cdot \mathbf{J}_{\mathrm{ex}} = G_{\mathrm{ex}} - \frac{n_{\mathrm{ex}}}{\tau_{\mathrm{ex}}}, \quad \text{where } \mathbf{J}_{\mathrm{ex}} = -D_{\mathrm{ex}} \nabla n_{\mathrm{ex}} \qquad \text{(Supplementary Equation 6)}$$

$$n_{\mathrm{ex}}|_{\text{domain boundary}} = 0, \quad \nabla n_{\mathrm{ex}}|_{\text{domain boundary}} = 0$$

where D_{ex} is the diffusivity, n_{ex} is the concentration, and τ_{ex} is the life time of the exciton. The exciton generation rate, G_{ex}, can be further calculated using a quantitative analysis of the steady-state distribution of E-field at given incident wavelength and power[7-9], as shown in Supplementary Equation 3. From Supplementary Equation 6, it is also possible to connect $G_{\mathrm{e/h}}$ and G_{ex} via such that[12,16]

$$G_{\mathrm{e/h}} = \frac{G_{\mathrm{ex}}(D_{\mathrm{ex}}\tau_{\mathrm{ex}})^{\frac{1}{2}} \cdot I_1\left[\frac{2\phi_{\mathrm{D}}H_{\mathrm{inter}}}{(D_{\mathrm{ex}}\tau_{\mathrm{ex}})^{\frac{1}{2}}}\right]}{H_{\mathrm{inter}} \cdot I_0\left[\frac{2\phi_{\mathrm{D}}H_{\mathrm{inter}}}{(D_{\mathrm{ex}}\tau_{\mathrm{ex}})^{\frac{1}{2}}}\right]} \qquad \text{(Supplementary Equation 7)}$$

where $I_0(x)$ and $I_1(x)$ are the zeroth- and first-order-modified Bessel function of the first kind, respectively. It is therefore found from Supplementary Equation 7 that G_{ex} monotonically decreases with increasing H_{inter}. From Supplementary Equation 5, we can find that the charge carrier transport dynamics is strongly affected by H_{inter}; too small H_{inter} gives rise to the higher bimolecular recombination rate, whereas too large H_{inter} results in the decrease in the generation rate of the electron-hole pairs at the donor-acceptor interface. Typically, H_{inter} is expected to greater than H_{PFM} by 10^1 - 10^2 times based on the reduced bimolecular recombination rate compared to the Langevin rate in the BHJ[11,15]. In order to find the fitting parameters in the governing equations (Supplementary Equations 5, 6, and 7), we tried to numerically solve the equations to find the best fit of J–V curve with calibrations of $k_{\mathrm{r}}, p, H_{\mathrm{PFM}}$, and D_{ex} under the assumed AM 1.5G conditions at room temperature. Calibration of the parameters used in the model was conducted by solving a nonlinear fitting in a least square manner. For the least square error, we found the parameters such that $k_{\mathrm{r}} = 5.36 \times 10^{-11}\ \mathrm{m^{-3}s^{-1}}, p = 0.90, H_{\mathrm{PFM}} = 1.96\ \mathrm{nm}$, and $D_{\mathrm{ex}} = 1.93 \times 10^{-7}\ \mathrm{m^2s^{-1}}$. We also employed the reported value for τ_{ex} such that $\tau_{\mathrm{ex}} = 4 \times 10^{-10}$ s[17]. With the calibrated parameters, we numerically calculated the photovoltaic performances (V_{OC}, J_{SC}, FF, and PCE) of the q- and b-BHJ OPVs. As apparent from Supplementary Figure 17, the parameters decreased with increasing H_{inter}, except the case of V_{OC}. Compared to other parameters, V_{OC} was nearly constant regardless of the H_{inter}, which can be explained by the V_{OC} being independent of the domain growth[18]. It is notable that the q-OPV is superior to the b-OPV in every manner of the photovoltaic parameters.

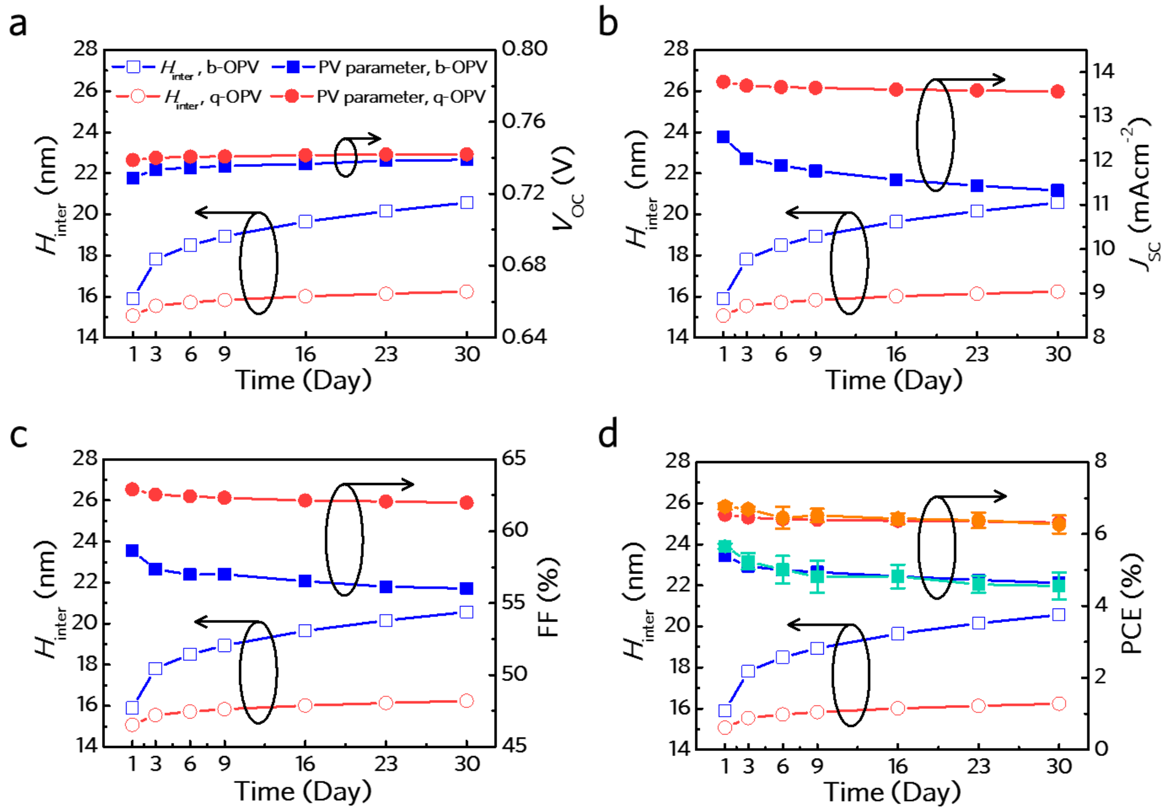

Supplementary Figure 18. Time-dependent H_{inter} and H_{inter}-derived photovoltaic parameters. Time-dependent (a) V_{OC}, (b) J_{SC}, (c) FF, and (d) PCE values of the b-OPV (solid blue square) and q-OPV (solid red circle) calculated from the H_{inter} (open blue square for b-OPV and open red circle for q-OPV) via employing the modified drift-diffusion and Poisson equations. The solid green square and solid orange circle in (d) indicate the experimentally measured PCEs of the b- and q-OPVs, respectively. The calculated PCE values nearly completely matched the experimental values.

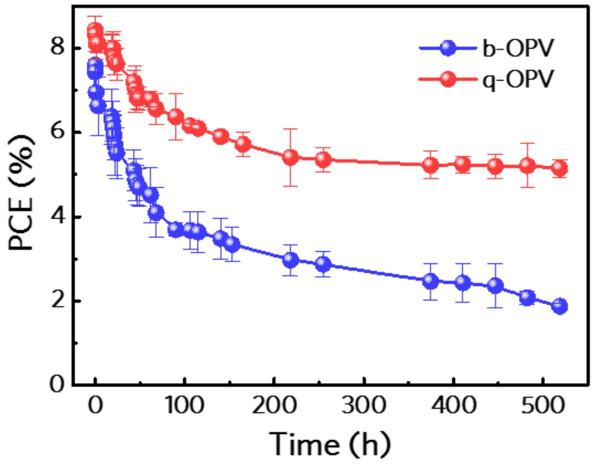

Supplementary Figure 19. Long-term photo-induced degradation test. Long-term PCE decay of b- and q-OPVs under AM 1.5G solar simulator with a 12 h light/dark illumination cycle for more than 21 days. The average and standard deviation values were obtained from at least 4 devices for each type of OPV.

Supplementary Table 7. Superior PCE sustainability of our q-OPV. Comparison of the PCE decay (the percentage of PCE decrease relative to the initial value) between our q-OPV and other state-of-the-art binary OPVs under diverse aging conditions (some PCE values estimated from the figure images, and not exactly stated in literature, are labeled with *ca*).

D:A material	Device structure	Initial PCE	Last PCE	PCE loss	Degradation conditions (*e.g.*, light, temperature, encapsulation)	Ref.
q-OPV	Inverted	8.42%	6.27% after 30 days (experimental) & 6.06% after one year (simulated)	25.53% & 28.03%	Dark, 65°C, with encapsulation	This study
b-OPV	Inverted	7.59%	4.56% after 30 days (experimental) & 3.39% after one year (simulated)	39.92% & 55.34%	Dark, 65°C, with encapsulation	This study
q-OPV	Inverted	8.42%	5.15% after ~21 days	38.84%	Illumination under AM 1.5G solar simulator (12 h light/dark illumination cycle), with encapsulation	This study
b-OPV	Inverted	7.59%	1.88% after ~21 days	75.23%	Illumination under AM 1.5G solar simulator (12 h light/dark illumination cycle), with encapsulation	This study
P3HT:$PC_{61}BM$	Standard	3.0%	*ca.* 1.5% after 4700h	*ca.* 50%	Continuous illumination under a Sulphur plasma lamp, 50°C (testing chamber temperature), with encapsulation	19
P3HT:$PC_{61}BM$	Standard	3.7%	2.5% after 1000h	32.43%	Dark, 45°C, w/o encapsulation (inert measurement conditions)	20
P3HT:$PC_{61}BM$	Standard	3.2%	1.8% after 1000h	43.75%	Continuous illumination under a 150W Xenon lamp with AM 1.5G filter, 45°C, w/o encapsulation (inert measurement conditions)	20
P3HT:$PC_{61}BM$	Standard	4.0 ± 0.05%	*ca.* 2.92% after 4400 h (experimental) & *ca.* 2.72% after 3.1 years (simulated)	27% & 32%	Continuous illumination under a Sulfur plasma lamp (6000 K), 37°C, with encapsulation	21
PCDTBT:$PC_{71}BM$	Standard	5.5 ± 0.15%	*ca.* 3.74% after 4400h (experimental) & *ca.* 3.19% after 6.2 years (simulated)	32% & 42%	Continuous illumination under a Sulfur plasma lamp (6000 K), 37°C, with encapsulation	21

$PCDTBT:PC_{71}BM$	Standard	7.04%	*ca.* 5.56% after 19500h (simulated) & *ca.* 5.63% after 650 days (experimental)	21.02% & 20.03%	Continuous illumination under a Sulphur plasma lamp (6000 K), room temperature, with encapsulation	22
$PCDTBT:PC_{71}BM$	Standard	6.50%	*ca.* 3.25% after 30 days	*ca.* 50%	Under ambient air conditions, w/o encapsulation	23
$PCDTBT:PC_{71}BM$	Standard	5.02%	3.54% after 4500h	29.48%	Continuous illumination under a halide lamp (1000 Wm^{-2}), 45°C, with encapsulation	24
$PTB7:PC_{71}BM$	Inverted	5.37%	*ca.* 3.33% after *ca.* 3500h	*ca.* 37.98%	Under ambient dark conditions, w/o encapsulation	25

Supplementary References

1. Cui, C., Wong, W. –Y. & Li, Y. Improvement of open-circuit voltage and photovoltaic properties of 2D-conjugated polymers by alkylthio substitution. *Energy Environ. Sci.* **7,** 2276-2284 (2014).

2. Razzell-Hollis, J., Wade, J., Tsoi, W. C., Soon, Y., Durrant, J. & Kim, J. –S. Photochemical stability of high efficiency PTB7:$PC_{70}BM$ solar cell blends. *J. Mater. Chem. A* **2,** 20189-20195 (2014).

3. Giannozzi, P. *et al.* Quantum ESPRESSO: A modular and open-source software project for quantum simulations of materials. *J. Phys.: Condens. Mater* **21,** 395502 (2009).

4. Gupta, V., Bharti, V., Kumar, M., Chand, S. & Heeger, A. J. Polymer-polymer Förster resonance energy transfer significantly boosts the power conversion efficiency of bulk-heterojunction solar cells. *Adv. Mater.* **27,** 4398-4404 (2015).

5. Huang, J. –S. *et al.* Polymer bulk heterojunction solar cells employing Förster resonance energy transfer. *Nature Photon.* **7,** 479-485 (2013).

6. Xiao, Y. *et al.* Efficient ternary bulk heterojunction solar cells with PCDTBT as hole-cascade material. *Nano Energy* **19,** 476-485 (2016).

7. Pettersson, L. A. A., Roman, L. S. & Inganäs, O. Modeling photocurrent action spectra of photovoltaic devices based on organic thin films. *J. Appl. Phys.* **86,** 487 (1999).

8. Peumans, P., Yakimov, A. & Forrest, S. R. Small molecular weight organic thin-film photodetectors and solar cells. *J. Appl. Phys.* **93,** 3693 (2003).

9. Burkhard, G. F., Hoke, E. T. & McGehee, M. D. *Adv. Mater.* **22,** 3293-3297 (2010).

10. Petoukhoff, C. E. & O’Carroll, D. M. Absorption-induced scattering and surface plasmon out-

coupling from absorber-coated plasmonic metasurfaces. *Nat. Commun.* **6**, 7899 (2015).

11. Maturova, K., van Bavel, S. S., Wienk, M. M., Janssen, R. A. J. & Kemerink, M. Morphological device model for organic bulk heterojunction solar cells. *Nano Lett.* **9,** 3032-3037 (2009).

12. Zhang, T., Birgersson, E. & Luther, J. A spatially smoothed device model for organic bulk heterojunction solar cells. *J. Appl. Phys.* **113,** 174505 (2013).

13. Balluffi, R. W., Allen, S. M. & Carter, W. C. Kinetics of Materials. Cambridge: Wiley-Interscience (2005).

14. Cook, H. Brownian motion in spinodal decomposition. *Acta Metall.* **18,** 297-306 (1970).

15. Zhu, J., Chen, L., Shen, J. & Tikare, V. Coarsening kinetics from a variable-mobility Cahn-Hilliard equation: application of a semi-implicit Fourier spectral method. *Phys. Rev. E* **60,** 3564 (1999).

16. Zhang, T., Birgersson, E. & Luther, J. Closed-form expressions correlating exciton transport and interfacial charge carrier generation with the donor/acceptor morphology in organic bulk heterojunction solar cells. *Physica B* **456,** 267-274 (2015).

17. Shaw, P. E., Ruseckas, A. & Samuel, I. D. W. Exciton diffusion measurements in poly(3-hexylthiphene). *Adv. Mater.* **20,** 3516-3520 (2008).

18. Ray, B., Nair, P. R. & Alam, M. A. Annealing dependent performance of organic bulk-heterojunction solar cells: A theoretical perspective. *Sol. Energy Mater. Sol. Cells* **95,** 3287-3294 (2011).

19. Zimmermann, B., Würfel, U. & Niggemann, M. Longterm stability of efficient inverted P3HT:PCBM solar cells. *Sol. Energy Mater. Sol. Cells* **93,** 491-496 (2009).

20. Voroshazi, E., Verreet, B., Aernouts, T. & Heremans, P. Long-term operational lifetime and degradation analysis of P3HT:PCBM photovoltaic cells. *Sol. Energy Mater. Sol. Cells* **95,** 1303-1307 (2011).

21. Peters, C. H. *et al.* High efficiency polymer solar cells with long operating lifetimes. *Adv. Energy Mater.* **1,** 491-494 (2011).

22. Kong, J. *et al.* Long-term stable polymer solar cells with significantly reduced burn-in loss. *Nature Commun.* **5,** 5688 (2014).

23. Sun, Y. *et al.* Efficient, air-stable bulk heterojunction polymer solar cells using MoO_x as the anode interfacial layer. *Adv. Mater.* **23,** 2226-2230 (2011).

24. Roesch, R., Eberhardt, K., Engmann, S., Gobsch, G. & Hoppe, H. Polymer solar cells with enhanced lifetime by improved electrode stability and sealing. *Sol. Energy Mater. Sol. Cells* **117,** 59-66 (2013).

25. Romero-Gomez, P. *et al.* Enhanced stability in semi-transparent PTB7/PC71BM photovoltaic cells. *Sol. Energy Mater. Sol. Cells* **137,** 44-49 (2015).
